# Results following implementation of a cardiac surgery ERAS protocol

**DOI:** 10.1371/journal.pone.0277868

**Published:** 2023-07-14

**Authors:** Tomi Obafemi, Danielle Mullis, Simar Bajaj, Purnima Krishna, Jack Boyd

**Affiliations:** Department of Cardiothoracic Surgery, Stanford University, Stanford, CA, United States of America; Saud Al-Babtain Cardiac Centre, SAUDI ARABIA

## Abstract

**Introduction:**

Adequate peri-operative care is essential to ensuring a satisfactory outcome in cardiac surgery. In this study, we look at the impact of evidence-based protocols implemented at Stanford Hospital.

**Methods:**

This study is a single-center, retrospective analysis. Enhanced recovery after surgery (ERAS) protocols were implemented for CABG/Valve and open Aortic operations on 11/1/2017 and 6/1/2018, respectively. Propensity-score matched analysis was used to compare 30-day mortality and morbidity of patients from the pre- and post-implementation cohorts. Secondary endpoints included the following: total hospital length of stay (LOS), ICU LOS, time until extubation, and time until urinary catheter removal.

**Results:**

After the implementation of the ERAS protocols for CABG/Valve operations, the median post-op LOS decreased from 7.0 days to 6.1 days (p<0.001), and median ICU LOS decreased from 69.9 hours to 54.0 (p = 0.098). There was no significant decrease in 30-day mortality (4% to 3.3%, p = 0.47). However, the incidence of post-op ventilator associated pneumonia (VAP) decreased from 5.0% to 2.1% (p = 0.003) and post-op urinary tract infections (UTIs) from 8.3% to 3.6% (p<0.001). Patients who underwent open aortic procedures experienced an improvement in 30-day mortality (7% to 3.5%, p = 0.012), decrease in median ICU LOS (91.7 hours to 69.6 hours, p<0.001), and a decrease in duration of mechanical ventilation (79.3 hours to 46.3 hours, p = 0.003). There was a decrease in post-op LOS, post-op VAP, and post-op UTI, although statistical significance was not attained.

**Conclusion:**

At Stanford Hospital, ERAS pathways have led to decreased morbidity and LOS while simultaneously improving mortality amongst our critically ill patient population.

## Introduction

Comprehensive perioperative care is essential to ensuring excellent surgical outcomes. Such care promotes recovery and has the potential to improve postoperative morbidity after cardiac surgery [1]. Successful initiatives rely on cohesive multidisciplinary interactions between surgical, anesthetic, and intensive care unit (ICU) teams [2]. Evidence-based enhanced recovery after surgery (ERAS) protocols provide guidelines for the creation of perioperative pathways intended to optimize patient care. These protocols also help to standardize care. Medical knowledge continues to evolve at an exponential rate [3]; to maintain a standard of quality care, cardiovascular and thoracic surgeons must be continually educated about evidence-based practices through the use of clinical guidelines and consensus documents.

A small pilot study by Fleming and colleagues in 2016 demonstrated promising outcomes for ERAS implementation in cardiac surgery. The study showed that ERAS protocols were feasible and had the potential to result in improved postoperative morbidity [4]. Despite these findings, adoption of enhanced recovery protocols has faced resistance in the field of cardiac surgery due to the complexity of the operations, acuity of the patient population, and lack of published evidence to support implementation. In 2019, cardiac surgery ERAS recommendations were published by the ERAS Cardiac Society with guidelines developed by a multidisciplinary group consisting of cardiac surgeons, anesthesiologists, and intensivists with previous experience with ERAS. This group agreed upon 22 potential interventions and divided them into the preoperative, intraoperative, and postoperative phases of recovery [5].

In previous literature, postoperative ERAS strategies have focused on early extubation, promotion of patient mobility, pain control, nutrition, gastrointestinal function, and fluid management [6].

At our institution, we have established comprehensive evidenced-based ERAS post-operative care pathways for CABG/Valve and Aortic surgeries. In this study, we looked at the impact of these evidence-based pathways. We compare quality metrics measured in the post pathway implementation cohort with a similar patient population that preceded the implementation of the ERAS pathways.

## Method

This study was a single-center retrospective analysis of consecutive patients undergoing Open Aortic, CABG, and Valve surgery at Stanford Hospital. The data was collected via the electronic medical record (EMR). The EMR utilized at Stanford Hospital during this time period was Epic Systems Corporations (Verona, WI). The study was approved by the Stanford IRB (#60785) and adhered to the institution’s protocols. The Stanford IRB waived the requirement for informed consent for this study and HIPAA guidelines was adhered to during the collection of data and reporting of our results.

The start dates for the CABG & Valve pathway and the Aortic Procedure pathway were 11/01/2017 and 06/01/2018, respectively. Order sets were created within Epic to implement the post-operative care pathways and standardize care. Orders are placed by the surgical team immediately after the completion of the procedure prior to leaving the operating room. The orders are subsequently released by the ICU team on the arrival of the patient to the unit.

In our analysis of the impact of the CABG & Valve pathway, we included patients who underwent isolated CABG cases, isolated Valve repair and replacement operations, and CABG/Valve combination procedures via robot-assisted, full sternotomy, partial sternotomy, and thoracotomy approaches. Patients who underwent transcatheter valve repair or replacement were excluded from the study as they were not managed postoperatively using the CABG & Valve order set. We use propensity scores to match patients into the pre-implementation and post-implementation groups with a cohort that underwent similar operations prior to the implementation of the pathway from 08/05/2009–11/02/2017. Patients were stratified into these two groups, and a logistic regression model was used to calculate propensity scores based on history of COPD, dialysis, diabetes, and NYHA score. We applied a greedy nearest neighbor matching algorithm without replacement with a caliper of 0.01 to calculate propensity scores. Balance of the match was assessed using standardized differences.

The analysis of the Aortic Procedure pathway included patients who underwent open aortic surgery after its implementation in June 2018. Open aortic surgery at our institution addresses a wide spectrum of aortic disease including aneurysms, dissections, and rupture; operations span the entirety of the thoracic aorta, from valve sparing root replacements to thoracoabdominal aneurysm repairs. Patients who had surgeries that utilized an endovascular approach (i.e., TEVAR) were excluded from our study. Patients on ECMO or those who had an intra-aortic balloon pump (IABP) were also excluded from our study. These patients were also propensity matched with patients who had similar procedures prior to the pathway implementation from 09/15/2012–06/01/2018. A logistic regression model was similarly used to calculate propensity scores among the pre-implementation group versus the post-implementation group based on history of COPD, diabetes, and NYHA score. Mean pain scores were obtained from post-operative day three. They were obtained from the patient directly, if extubated. The scale used ranged from 0 to 10. 0 was considered no pain at all and 10 was considered to be the worst pain imagined.

**Fig 1:**
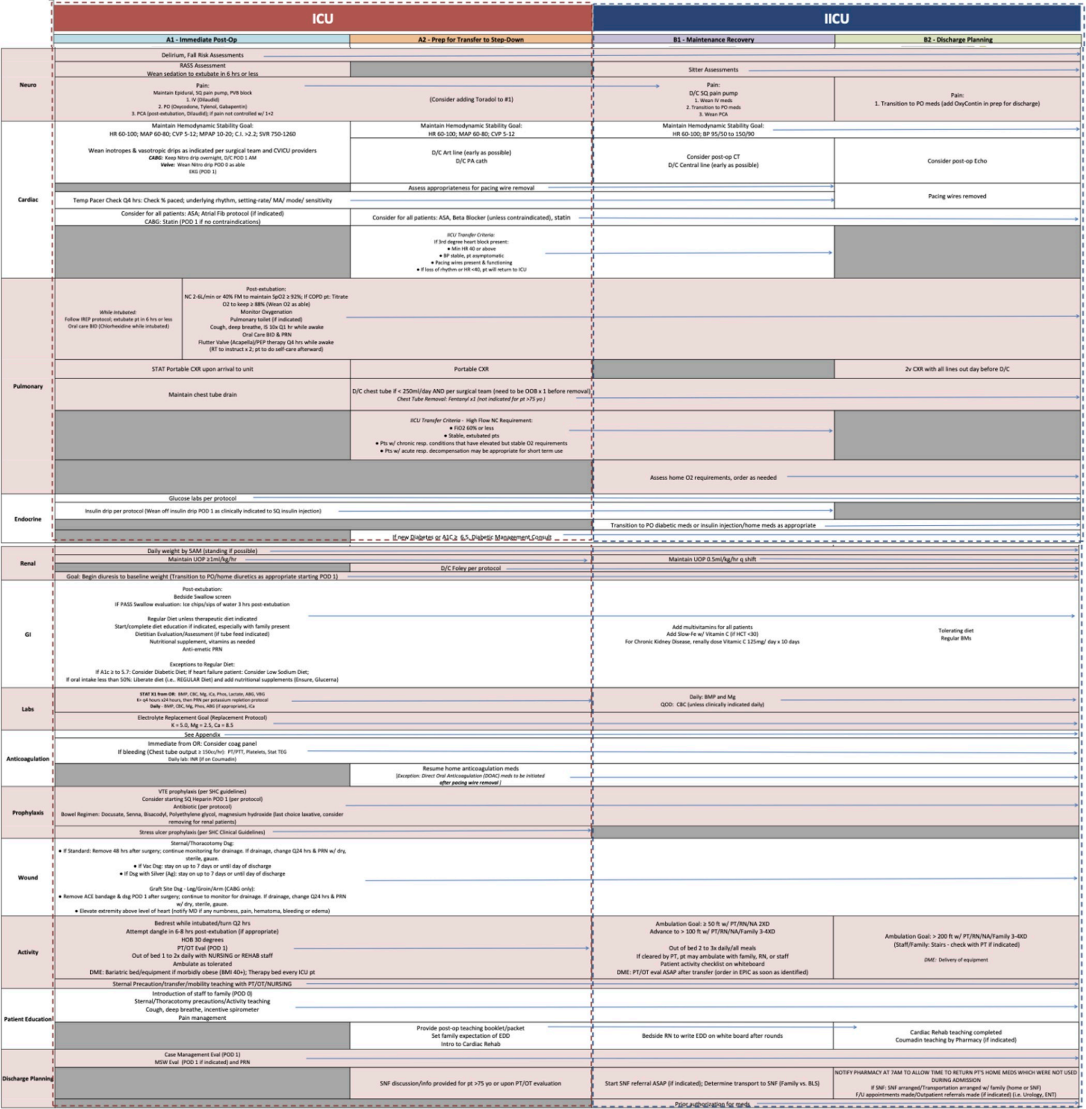
A breakdown of the CABG/Valve procedure post op care pathways. The comprehensive evidence-based post-operative care pathways at our institution adopt a multimodal approach and are broken down into organ systems: neurological, cardiac, pulmonary, endocrine, renal, gastrointestinal, labs, anticoagulation, prophylaxis, wound, activity, patient education, and discharge planning.

**Fig 2:**
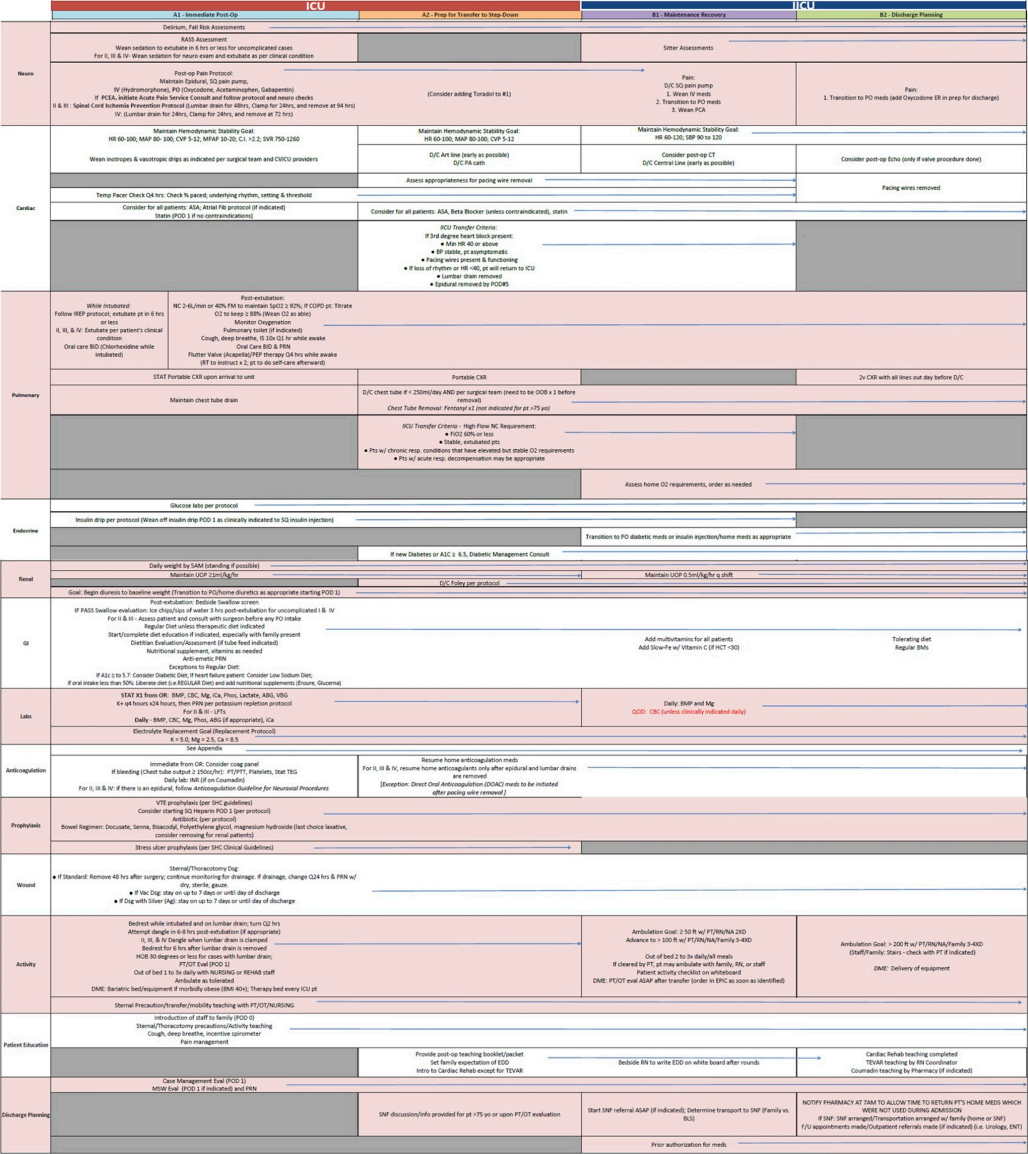
A breakdown of Aortic procedures post op care pathways. The comprehensive evidence-based post-operative care pathways at our institution adopt a multimodal approach and are broken down into organ systems: neurological, cardiac, pulmonary, endocrine, renal, gastrointestinal, labs, anticoagulation, prophylaxis, wound, activity, patient education, and discharge planning.

The comprehensive evidence-based post-operative care pathways at our institution adopt a multimodal approach and are broken down into organ systems: neurological, cardiac, pulmonary, endocrine, renal, gastrointestinal, labs, anticoagulation, prophylaxis, wound, activity, patient education, and discharge planning. Figs 1 & 2 depict a complete breakdown of the CABG/Valve and Aortic procedure post op care pathways. Main components of the protocol were early extubation, ambulation, tight hemodynamic control, multimodal pain control and pre-emptive discharge planning. The primary endpoint of the study was impact of the post-op pathway on 30-day mortality and morbidity (UTIs, VAP, Wound infections). The secondary endpoints evaluated were total hospital length of stay (LOS), ICU LOS, time until extubation, time until first ambulation, time until urinary catheter removal, time until initiation of enteral feeding, and duration of mechanical ventilation.

## Results

In the CABG/Valve group, 1131 patients were included in the pre-implementation cohort and 756 in the post-implementation cohort (Table 1). In the Aortic group, 601 patients were included in the pre-implementation cohort and 833 in the post-implementation cohort (Table 2). Prior to matching, there was no difference in age, race, or gender in the CABG/Valve group. The Euroscore between the pre- and post-implementation groups was similar. We observed a significant improvement in median post op LOS from 7.0 days to 6.1 day between the two groups (p<0.001). The ICU LOS also decreased from 70.2 hours to 56.4 hours (p = 0.002). There were similar improvements in duration of mechanical ventilation, time to urinary catheter removal, day to first ambulation, days until first bowel movement, incidence of postop ventilator associated pneumonia (VAP), and post op urinary tract infections (Table 1). In the Aortic cohort, the age, race, and gender were also comparable prior to propensity matching. The Euroscore between the pre- and post-implementation groups was also similar in this cohort. There was an improvement in 30-day mortality from 7.2% to 3.1%. Again, we observed an improvement in median postop LOS from 8.4 days to 7.8 days (p = 0.041). The ICU LOS also improved from 91.8 hours to 68.6 hours (p<0.001). Duration of mechanical ventilation, time to urinary catheter removal, days to first ambulation, and incidence of postop UTIs all saw improvements as well (Table 2).

**Table 1:** Pre-match CABG/Valve group.

Patient Characteristic	Pre-Implementation	Post-Implementation	P-Value
	1131	756	
Age at Procedure	67.5 (59.6, 75.0)	68.6 (60.9, 74.5)	0.27
Female Gender, n(%)	259 (22.9%)	159 (21.0%)	0.34
Race, n(%)			0.88
White	646 (57.1%)	411 (54.4%)	
Black	29 (2.6%)	18 (2.4%)	
Native American	5 (0.4%)	4 (0.5%)	
Pacific Islander	20 (1.8%)	14 (1.9%)	
Asian	220 (19.5%)	154 (20.4%)	
Other/Unknown	211 (18.7%)	155 (20.5%)	
Pre-Op COPD, n (%)	110 (9.7%)	50 (6.6%)	0.017
Pre-Op Dialysis, n(%)	50 (4.4%)	52 (6.9%)	0.021
Pre-Op Diabetes, n(%)	474 (41.9%)	405 (53.6%)	<0.001
History of Atrial Fibrillation, n(%)	120 (10.6%)	101 (13.4%)	0.069
Pre-Op HTN, n(%)	904 (79.9%)	598 (79.1%)	0.66
History of Ventricular Tachycardia, n(%)	19 (1.7%)	21 (2.8%)	0.10
NYHA, n(%)			<0.001
0	507 (44.8%)	277 (36.6%)	
1	547 (48.4%)	464 (61.4%)	
2	49 (4.3%)	6 (0.8%)	
3	22 (1.9%)	7 (0.9%)	
4	6 (0.5%)	2 (0.3%)	
Post-Op LOS day (IQR)	7.0 (5.2, 11.2)	6.1 (4.9, 8.8)	<0.001
ICU LOS hours (IQR)	70.2 (42.8, 118.0)	56.4 (40.6, 99.2)	0.002
Post-Op Vent hours (IQR)	286.1 (31.9, 844.5)	23.9 (9.6, 127.4)	<0.001
Days to Urinary Catheter Removal (IQR)	2.6 (1.8, 4.0)	1.9 (1.2, 3.0)	<0.001
Days to First Ambulation (IQR)	2.4 (1.6, 3.5)	1.6 (1.5, 2.6)	<0.001
Days to First Bowel Movement (IQR)	3.0 (2.0, 4.0)	2.0 (0.0, 3.0)	<0.001
Post-Op Ventilator Associated Pneumonia	58 (5.1%)	17 (2.2%)	0.002
30-day mortality	43 (3.8%)	26 (3.4%)	0.68
Post-Op Urinary Tract Infection	99 (8.8%)	27 (3.6%)	<0.001
Post-Op Wound Infection	7 (0.6%)	1 (0.1%)	0.11
Euroscore (IQR)	2.0 (1.0, 3.9)	1.7 (1.0, 3.5)	0.084

Pre-match CABG/Valve Group. Values are presented as n (%) or median (interquartile range).

**Table 2:** Pre-match open aortic group.

Patient Characteristic	Pre-Implementation	Post-Implementation	P-Value
	601	833	
Age at Procedure	61.6 (50.3, 71.8)	62.1 (50.8, 70.7)	0.68
Female Gender, n(%)	180 (30%)	218 (26.2%)	0.11
Race, n(%)			0.71
White	379 (63.1%)	524 (62.9%)	
Black	41 (6.8%)	48 (5.8%)	
Native American	3 (0.5%)	1 (0.1%)	
Pacific Islander	7 (1.2%)	10 (1.2%)	
Asian	70 (11.6%)	108 (13.0%)	
Other/Unknown	101 (16.8%)	142 (17.0%)	
Pre-Op COPD, n (%)	40 (6.7%)	38 (4.6%)	0.085
Pre-Op Dialysis, n(%)	7 (1.2%)	16 (1.9%)	0.26
Pre-Op Diabetes, n(%)	59 (9.8%)	118 (14.2%)	0.014
History of Atrial Fibrillation, n(%)	74 (12.3%)	86 (10.3%)	0.24
Pre-Op HTN, n(%)	354 (58.9%)	489 (58.7%)	0.94
History of Ventricular Tachycardia, n(%)	8 (1.3%)	9 (1.1%)	0.67
NYHA, n(%)			0.003
0	416 (69.2%)	495 (59.4%)	
1	177 (29.5%)	324 (38.9%)	
2	3 (0.5%)	5 (0.6%)	
3	4 (0.7%)	9 (1.1%)	
4	1 (0.2%)	0 (0.0%)	
Post-Op LOS day (IQR)	8.4 (5.9, 13.8)	7.8 (5.8, 12.5)	0.041
ICU LOS hours (IQR)	91.8 (50.7, 139.0)	68.6 (45.0, 118.9)	<0.001
Post-Op Vent hours (IQR)	78.9 (16.6, 750.1)	40.3 (13.2, 249.4)	<0.001
Days to Urinary Catheter Removal (IQR)	2.9 (1.8, 4.8)	2.3 (1.6, 3.7)	<0.001
Days to First Ambulation (IQR)	2.5 (1.6, 3.6)	1.7 (1.5, 3.2)	<0.001
Days to First Bowel Movement (IQR)	3.0 (1.0, 4.0)	3.0 (1.0, 3.0)	<0.001
Post-Op Ventilator Associated Pneumonia	51 (8.5%)	51 (6.1%)	0.086
30-day mortality	43 (7.2%)	26 (3.1%)	<0.001
Post-Op Urinary Tract Infection	40 (6.7%)	26 (3.1%)	0.002
Post-Op Wound Infection	8 (1.3%)	9 (1.1%)	0.67
Euroscore (IQR)	3.1 (1.7, 5.5)	2.9 (1.6, 5.9)	0.76

Pre-match Open Aortic Group. Values are presented as n (%) or median (interquartile range).

A total of n = 747 patients from the CABG/Valve pre-implementation group and CABG/Valve post-implementation groups were propensity matched, as seen in Table 3. There were no significant differences between the demographic characteristics, comorbidities, or NYHA class of either group after matching. Between the two matched groups, there was a significant difference in the post-op length of stay, with the median LOS for the pre-implementation group being 7.0 days and the post-implementation group averaging 6.0 days (p<0.001). The median ICU LOS decreased from 69.9 hours to 54.0 hours in the post-implementation group (p<0.001). Patients in the post-implementation group also experienced a decreased incidence of postop UTIs (8.3% to 3.6%, p<0.001), faster return to ambulation after surgery (2.3 days vs 1.6 days, p = 0.001), and a shorter time to first bowel movement (3.0 days vs 2.0 days, p<0.001). The median duration of mechanical ventilation decreased from 272.4 hours to 23.5 hours (p<0.001). There was no significant decrease in 30-day mortality (4% to 3.3%, p = 0.47), time to urinary catheter removal (2.4 days to 2.3 days, p = 0.82), and post-op wound infections (0.4% to 0.1%, p = 0.32) (Table 3).

**Table 3:** Propensity-score matched CABG/Valve group.

Patient Characteristic	Pre-Implementation	Post-Implementation	P-Value
	747	747	
Age at Procedure	67.3 (59.4, 74.9)	68.7 (60.9, 74.6)	0.16
Female Gender, n(%)	165 (22.1%)	157 (21.0%)	0.61
Race, n(%)			0.98
White	409 (54.8%)	410 (54.9%)	
Black	21 (2.8%)	17 (2.3%)	
Native American	4 (0.5%)	4 (0.5%)	
Pacific Islander	15 (2.0%)	13 (1.7%)	
Asian	145 (19.4%)	152 (20.3%)	
Other/Unknown	153 (20.5%)	151 (20.2%)	
Pre-Op COPD, n (%)	47 (6.3%)	50 (6.7%)	0.75
Pre-Op Dialysis, n(%)	43 (5.8%)	43 (5.8%)	1.00
Pre-Op Diabetes, n(%)	394 (52.7%)	396 (53.0%)	0.92
History of Atrial Fibrillation, n(%)	83 (11.1%)	98 (13.1%)	0.23
Pre-Op HTN, n(%)	606 (81.1%)	589 (78.8%)	0.27
History of Ventricular Tachycardia, n(%)	11 (1.5%)	20 (2.7%)	0.10
NYHA, n(%)			1.00
0	276 (36.9%)	275 (36.8%)	
1	456 (61.0%)	457 (61.2%)	
2	6 (0.8%)	6 (0.8%)	
3	7 (0.9%)	7 (0.9%)	
4	2 (0.3%)	2 (0.3%)	
Post-Op LOS day (IQR)	7.0 (5.2, 11.0)	6.0 (4.9, 8.8)	<0.001
ICU LOS hours (IQR)	69.9 (40.8, 116.7)	54.0 (40.4, 97.0)	0.010
Post-Op Vent hours (IQR)	272.4 (22.2, 839.9)	23.5 (9.6, 122.6)	<0.001
Days to Urinary Catheter Removal (IQR)	2.4 (1.6, 3.9)	2.3 (1.6, 3.9)	0.82
Days to First Ambulation (IQR)	2.3 (1.6, 3.5)	1.6 (1.5, 2.6)	<0.001
Days to First Bowel Movement (IQR)	3.0 (2.0, 4.0)	2.0 (0.0, 3.0)	<0.001
Post-Op Ventilator Associated Pneumonia	37 (5.0%)	16 (2.1%)	0.003
30-day mortality	30 (4%)	25 (3.3%)	0.47
Post-Op Urinary Tract Infection	62 (8.3%)	27 (3.6%)	<0.001
Post-Op Wound Infection	3 (0.4%)	1 (0.1%)	0.32
Euroscore (IQR)	2.0 (1.0, 3.8)	1.6 (0.9, 3.4)	0.075

Propensity-Score Matched CABG/Valve Group. Values are presented as n (%) or median (interquartile range).

586 patients were matched in the Aortic pre- and post- implementation cohorts (Table 4). Compared to the pre-implementation group, there was a decrease in 30-day mortality from 7% to 3.5% (p = 0.012) in the post-implementation group. In contrast to the CABG/Valve cohort, the decrease in post-op LOS did not reach the level of significance (8.5 days vs 7.9 days, p = 0.32) in the post-implementation group, but ICU LOS decreased from 91.7 hours to 69.6 hours (p<0.001). In the post-implementation group, there was also a decrease in the duration of mechanical ventilation decreased from 69.7 hours to 35.2 hours, (p<0.001) and a faster return to ambulation after surgery (2.5 days vs 1.8 days, p = 0.001). Patients in the post-implementation group also experienced a decreased incidence of postop UTIs (6.3% to 3.9%, p = 0.064) and post-op VAP (8.5% to 6.1%, p = 0.12) although neither reached the level of significance. There was no decrease in time to urinary catheter removal (2.3 days to 2.4 days, p = 0.08) and post-op wound infections (1.4% to 1.2%, p = 0.79) (Table 4). Mean post-op day 3 pain scores in the matched post-implementation CABG/Valve cohort was 1.0 vs 1.2 (P = 0.008) when compared to the pre-ERAS cohort. In the Aortic group mean post-op day 3 pain scores were 0.8 vs 1.2 (p<0.001), respectively.

**Table 4:** Propensity-score matched open aortic group.

Patient Characteristic	Pre-Implementation	Post-Implementation	P-Value
	586	586	
Age at Procedure	61.5 (50.1, 71.7)	61.8 (50.6, 70.8)	0.71
Female Gender, n(%)	173 (29.5%)	173 (29.5%)	1.00
Race, n(%)			0.85
White	370 (63.1%)	356 (60.8%)	
Black	38 (6.5%)	42 (7.2%)	
Native American	3 (0.5%)	1 (0.2%)	
Pacific Islander	7 (1.2%)	9 (1.5%)	
Asian	69 (11.8%)	73 (12.5%)	
Other/Unknown	99 (16.9%)	105 (17.9%)	
Pre-Op COPD, n (%)	27 (4.6%)	27 (4.6%)	1.00
Pre-Op Dialysis, n(%)	7 (1.2%)	11 (1.9%)	0.34
Pre-Op Diabetes, n(%)	58 (9.9%)	73 (12.5%)	0.16
History of Atrial Fibrillation, n(%)	71 (12.1%)	59 (10.1%)	0.26
Pre-Op HTN, n(%)	344 (58.7%)	327 (55.8%)	0.32
History of Ventricular Tachycardia, n(%)	8 (1.4%)	7 (1.2%)	0.79
NYHA, n(%)			0.076
0	403 (68.8%)	423 (72.2%)	
1	176 (30.0%)	154 (26.3%)	
2	3 (0.5%)	0 (0.0%)	
3	4 (0.7%)	9 (1.5%)	
4	0 (0.0%)	0 (0.0%)	
Post-Op LOS day (IQR)	8.5 (5.9, 13.8)	7.9 (5.9, 13.0)	0.32
ICU LOS hours (IQR)	91.7 (50.6, 139.4)	69.6 (45.7, 127.9)	<0.001
Post-Op Vent hours (IQR)	79.3 (16.5, 740.3)	46.3 (13.2, 308.6)	0.003
Days to Urinary Catheter Removal (IQR)	2.3 (1.5, 3.8)	2.4 (1.6, 4.3)	0.080
Days to First Ambulation (IQR)	2.5 (1.6, 3.6)	1.8 (1.5, 3.4)	<0.001
Days to First Bowel Movement (IQR)	3.0 (1.0, 4.0)	3.0 (1.0, 3.0)	0.003
Post-Op Ventilator Associated Pneumonia	50 (8.5%)	36 (6.1%)	0.12
30-day mortality	41 (7%)	21 (3.6%)	0.012
Post-Op Urinary Tract Infection	37 (6.3%)	23 (3.9%)	0.064
Post-Op Wound Infection	8 (1.4%)	7 (1.2%)	0.79
Euroscore (IQR)	3.0 (1.6, 5.3)	3.2 (1.7, 5.7)	0.63

Propensity-Score Matched Open Aortic Group. Values are presented as n (%) or median (interquartile range).

## Discussion

The ERAS concept was first introduced by academic surgeons aiming to improve perioperative care for patients receiving colorectal care [7]; it is now practiced in almost all surgical fields. ERAS pathways help optimize patient care and are often used to describe a multimodal perioperative care program [8]. They standardize hospital workflows and ensure a standard of consistency and quality [9–11]. Studies have shown that ERAS protocols have been associated with a reduction in complication rates and LOS by up to 50% in general surgery patient populations [12].

Successful cardiac surgery requires cohesive integration of a sizeable team’s workflows in all phases of care. Post-operatively, it is imperative that the surgical team works in concert with the clinical staff in the ICU and in step-down units to enhance patient care. Standardized, evidence-based protocols that address, glycemic control, opioid-sparing pain management, post-operative nausea and vomiting (PONV), timely lines and catheter removal, extubation strategies, and early enteral feeding reduce surgical morbidity and have been shown to result in a reduced total hospital LOS, ICU LOS [1, 4].

At our institution, we have created separate evidenced-based care pathways for post-op CABG/Valve patients and patients who have undergone open aortic surgery. We excluded all patients who underwent endovascular procedures (i.e., TAVR, TEVAR), or required ECMO or an Intra-Aortic Balloon Pump. The post-op care pathways are broken down into 13 components: Neurological, Cardiac, Pulmonary, Endocrine, Renal, Gastrointestinal, Labs, Anticoagulation, Prophylaxis, Wound, Activity, Patient Education, and Discharge planning. The care pathways focus on earlier extubation when appropriate, pain control, delirium management, expedient attempts at ambulation, early swallow screen after extubation, enteral nutrition, aggressive bowel regimen, and DVT prophylaxis. Prior to the ERAS order set, although there was a focus to reduce opioid utilization there was no standardization in the multi-modal pain control regimen, stool softener regimen, ambulation or extubation protocol.

Our study showed significant improvement in both postoperative LOS and total ICU LOS in patients undergoing CABG/Valve and open Aortic procedures post protocol implementation. Significant improvements were also made in duration of mechanical ventilation, time to foley catheter removal, time to first ambulation, and time to first bowel movement for both groups as well. These findings are in line with previous studies [13, 14].

Past literature has shown an association of early extubation with decreased pulmonary complications and decreased LOS [2, 5]. Our results echoed these findings. The early extubation protocol at our institution facilitates tracheal extubation within 6 hours of surgery if the patient meets the criteria. Patients in the post-implementation cohort had a decreased duration of mechanical ventilation and overall fewer occurrences of ventilator associated pneumonia. Early extubation within 6 hours of surgery has been shown to be safe and is a class IIa, level B recommendation [2].

Prolonged indwelling urinary catheters are associated with increased risk of UTIs and surgical morbidity [15, 16]. Our care pathway utilizes a foley catheter removal protocol that facilitates a timely removal of the urinary catheter. Although our study did not show a decrease in time to catheter removal there was a decrease in the incidence of UTIs in both the Aortic and CABG/Valve groups.

Early ambulation decreases the incidence of atelectasis and improves patient recovery [17]. Once extubated our protocol calls for patients to be dangled on the edge of their bed, the same day, and assessed by the bedside nurse and a physical therapist for mobility. Patients are provided strength and mobility exercises and gradually increase walking distance in subsequent days. Our post-implementation CABG/Valve and Aortic cohorts demonstrated a shorter time to first ambulation.

An aggressive bowel regimen and timely resumption of enteral nutrition has been shown to be associated with a faster return of bowel function and healing [18, 19]. Once extubated patients at our center are given a bedside swallow assessment by a nurse or speech and linguistic therapist and started on a bowel regimen. The patient is progressed to an oral diet if there are no concerns for aspiration during the assessment. Patients in both post-implementation groups had shorter times to return of bowel function.

It is important to recognize the synergistic impact one aspect of the care pathway may have on another. An early extubation protocol allows for a probable reduction in the total dose of opioids and is associated with a decreased incidence of ventilator associated pneumonia. Early extubation has a direct impact on the timing of patient ambulation and allows for consideration of timely urinary catheter removal. Furthermore, return of bowel function has been linked to ambulation and decreased usage of opioids. Fortunately, the COVID pandemic did not result in any staffing constraints within our department. We have not had to change any of our intra-op or ICU post op protocols as a result of the pandemic. Pre-operatively we have added COVID testing to be in compliance with California state mandates.

## Conclusion

The implementation of the ERAS protocol at Stanford Hospital is associated with improvements in patient outcome and ensures patients will receive multi-modal care. Significant improvements in morbidity quality metrics can be expected to improve bed utilization and cost-revenue ratios.

Further investigation is warranted regarding the cost analysis of these post-op care pathways.
